# Detecting RNA modification using direct RNA sequencing: A systematic review

**DOI:** 10.1016/j.csbj.2022.10.023

**Published:** 2022-10-21

**Authors:** Xichen Zhao, Yuxin Zhang, Daiyun Hang, Jia Meng, Zhen Wei

**Affiliations:** a Department of Biological Sciences, Xi'an Jiaotong-Liverpool University, 215123 Suzhou, Jiangsu, China; b AI University Research Centre, Xi’an Jiaotong-Liverpool University, 215123 Suzhou, Jiangsu, China; c Institute of Systems, Molecular and Integrative Biology, L69 7ZB Liverpool, UK; d Institute of Life Course and Medical Sciences, L69 7ZB Liverpool, UK; e Department of Computer Science, University of Liverpool, L69 7ZB Liverpool, UK

## Abstract

Post-transcriptional RNA modifications are involved in a range of important cellular processes, including the regulation of gene expression and fine-tuning of the functions of RNA molecules. To decipher the context-specific functions of these post-transcriptional modifications, it is crucial to accurately determine their transcriptomic locations and modification levels under a given cellular condition. With the newly emerged sequencing technology, especially nanopore direct RNA sequencing, different RNA modifications can be detected simultaneously with a single molecular level resolution. Here we provide a systematic review of 15 published RNA modification prediction tools based on direct RNA sequencing data, including their computational models, input–output formats, supported modification types, and reported performances. Finally, we also discussed the potential challenges and future improvements of nanopore sequencing-based methods for RNA modification detection.

## Graphical abstract

**Figure:**
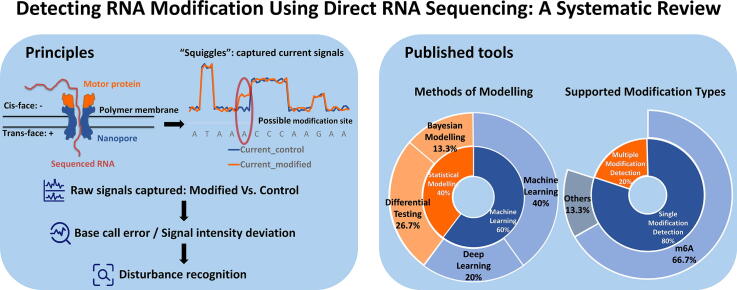

## 1 Introduction

Covalent modification of RNA has recently emerged as a critical layer of gene expression regulation in living cells [1]. RNA epitranscriptomic modifications can specify the metabolism fates of RNA molecules, such as the turnover rate of the RNA molecules [2], [3], the interaction between RNA and proteins [4], and translational accuracy and efficiency [5], [6]. To date, >140 types of RNA modifications have been identified on various types of cellular RNAs, including mRNA, tRNA, rRNA, and lincRNA [7]. Among them, N6-methyladenosine (m^6^A) is the most abundant modification in eukaryotic mRNAs. m^6^A modifications are mainly installed by the m^6^A methyltransferase complex comprised of METTL3, METTL14, and WTAP [8]. The internal m^6^A modification has proven to influence fundamental cellular processes, including the regulation of RNA decay [3], [9], translation efficiency [10] and splicing [11]. Recent studies demonstrated that both over and under methylation of m^6^A can lead to or accelerate tumor development [12], [13]. Similar to m^6^A, m^1^A modifications are methylated by protein complexes, such as TRMT6-TRMT61A [14]. m^1^A has been shown to affect the RNA 2ndary structures by disturbing the Watson–Crick base pairing through the positive charge on the modification site [15]. Pseudouridine (ψ) is another commonly identified RNA modification produced from either the RNA-dependent or RNA-independent pseudouridylation processes. ψ has been proposed to regulate the splicing and translation of mRNA [16]. It can also universally form pairs with A, U, G, and C bases, increasing the stability of RNA structures [17], [18]. 2′-O-methylation (Nm) is a highly conserved RNA modification occurring on any nucleotide across various RNA species [19]. Nm modifications are known to regulate the mRNA and protein expression, specifically via the assistance of small nucleolar RNAs [20].

To better understand the functional roles of RNA modifications under various conditions, it is important to accurately predict their genomic sites and modification levels for a given biological sample. Most existing methods for RNA modification profiling are based on 2nd generation sequencing technology that requires reverse transcription, in which the RNA modification information is removed from the sequencing signal. Therefore, either antibody enrichment or specific chemical treatment was combined with the standard NGS procedure to selectively identify RNA molecule modification sites [21]. These methods, though widely used, bear some technical defects, including the PCR enrichment bias, difficulty in finding antibodies and chemicals with high specificities, and lack of single-read level detection capacities [21], [22]. Thus, the 3rd generation sequencing technology has emerged as a promising novel technique to overcome these limitations.

**Fig. 1:**
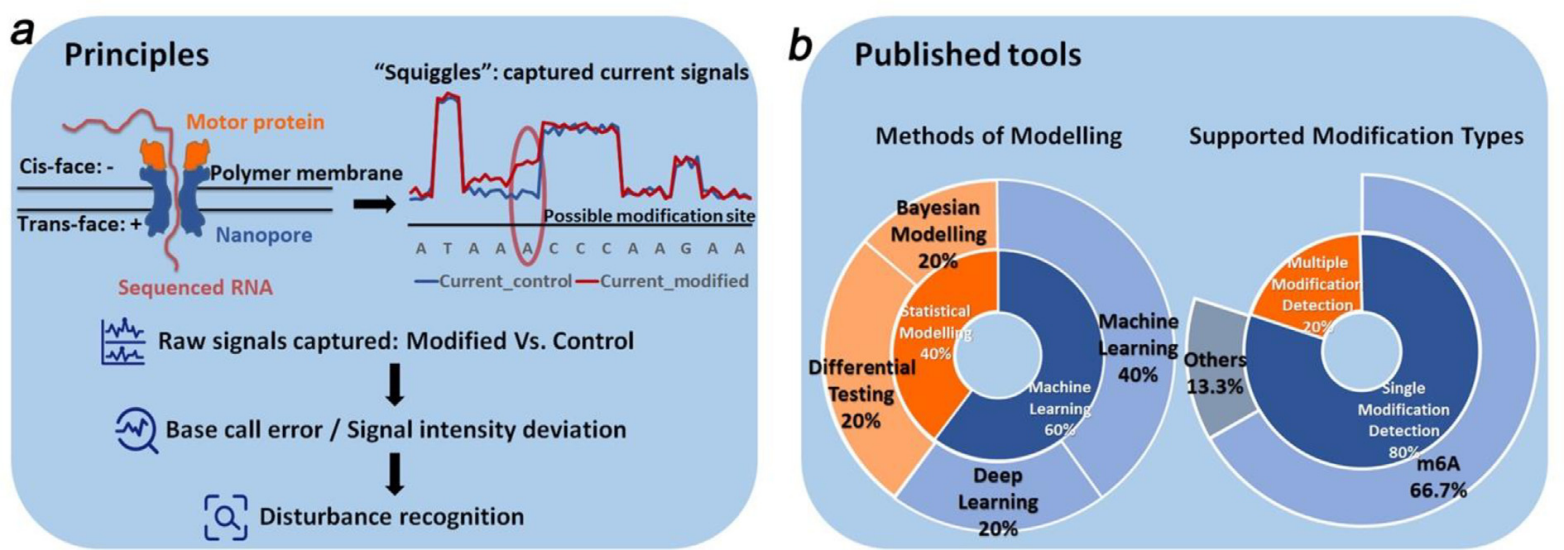
Illustration of nanopore sequencing principle. **a)**. The translocation of an RNA molecule will disturb the currents across the membrane, which are captured and recorded as “squiggles.” Published tools for RNA modification calling apply computational models to capture the deviation of the current signal in the modified base from the unmodified base. **b)**. Different computational strategies such as Bayesian models and deep learning were realized in corresponding pipelines to achieve this purpose. Most existing tools are trained on data of m^6^A and can identify only m^6^A modification.

Nanopore sequencing technology and its supporting nanopore sequencer MinION were developed and provided by Oxford Nanopore Technology (ONT) [23]. Compared to NGS, nanopore sequencing does not require reverse transcription, and the modification information is retained on single-read level data. In addition, nanopore sequencing is a portable device that can detect signals in real-time. Nanopore sequencing consists of three major components: the polymer membrane, the nanoscale protein pore embedded in the membrane, and the motor protein on the pore [24], [25]. While the sequencing is initiated, a voltage potential is kept constant across the membrane. The helicase motor protein will first unwind the DNA into a negatively charged single-stranded molecule (while the single-stranded RNA can be sequenced directly). Next, the nucleic acid is ratcheted from the negatively charged *cis*-face to the positively charged *trans*-face of the membrane through the nanopore (Fig. 1) [24], [25]. This translocation will lead to the alteration of voltage across the membrane and disrupt the currents [24], [25], [26], [27]. Since the degree of shift of the current signals is uniquely associated with the current nucleotide and its 5-mer sequence context, the captured changes of currents can be “basecalled”, i.e. to convert ionic signals into sequence information.

The base calling procedure is mainly realized through deep learning methods such as the recurrent neural network (RNN) [24], [25], [27]. The current signals are first segmented into separate “events”. An RNN takes an input of a sequence of vectors containing current values of these events and incorporates with the hidden state vectors from the previously hidden layers to produce an output of a sequence of vectors providing a probability distribution for each base [28], [29]. To consider the context information before and after the called base, bidirectional recurrent neural networks are applied in Guppy [28], [29].

Beyond the primary sequences, the nucleotide modification sites can be predicted directly from the read-level electrical signal by identifying the “deviant” from the regular signal shifts of the nucleotide (Fig. 1). To implement this procedure, current signal intensity levels are often extracted with software such as Nanopolish or Tombo to acquire a dataset containing signal statistics associated with base calling events [30]. Currently, many software tools have been developed to predict RNA modifications based on nanopore sequencing data, such as EpiNano [21], [31], nano-ID [32], DiffErr [33], MINES [34], Nanocompore [35], ELIGOS [36], xPore [26], nanom6A [37], nanoRMS [38], DRUMMER [39], nanoDoc [40], Yanocomp [41], Penguin [42], m6Anet [43] and DENA [44]. In this review article, we will first summarize the computational strategies used when developing the above tools. Following that, we try to contrast their functionalities by enumerating the supported modification types, the format of the input information, and their reported performances. Therefore, these efforts may help to find avenues to improve future development in computational methods and sequencing devices.

## 2 Review of methodologies

### 2.1 Statistical methods

**Fig. 2:**
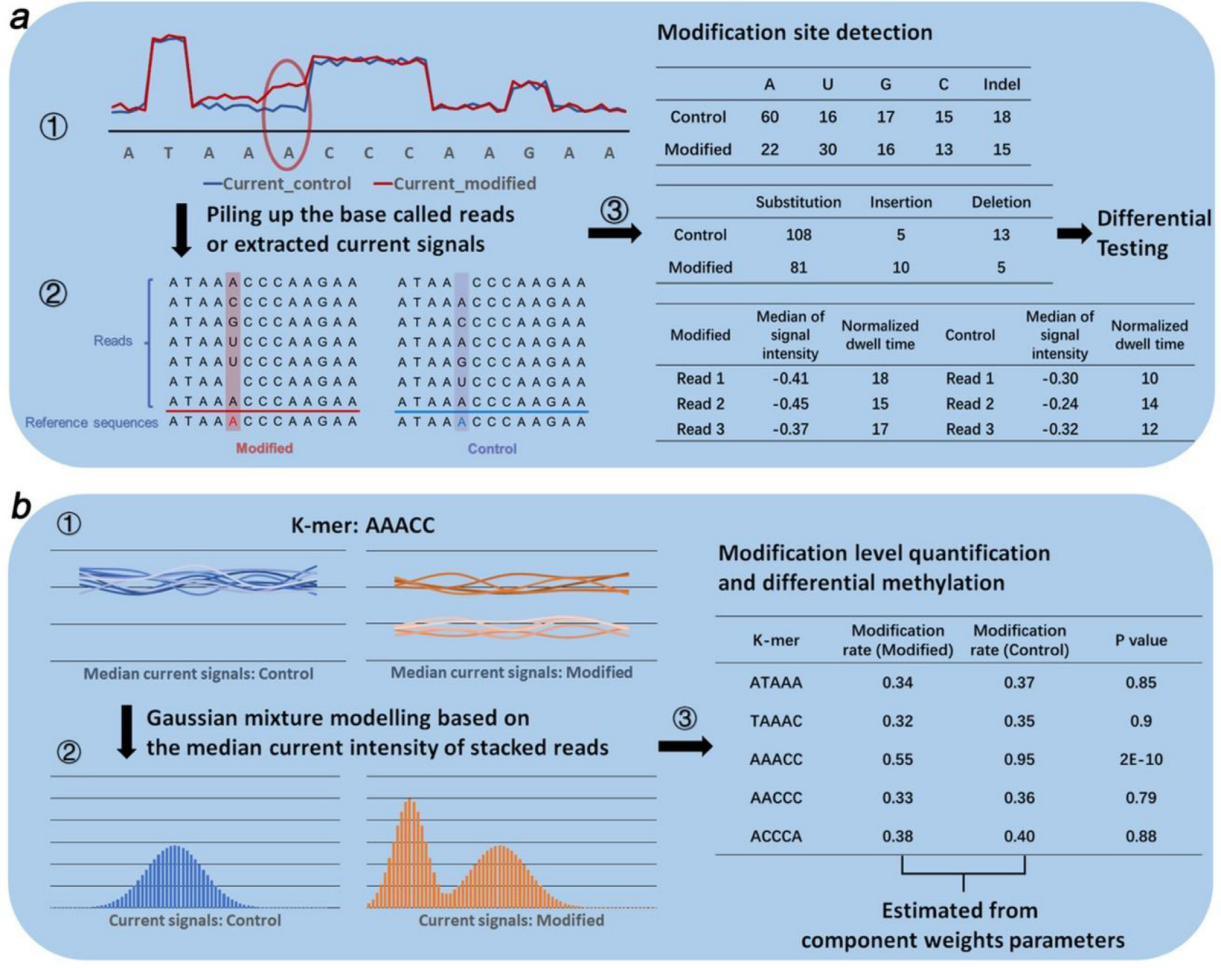
Two major computational frameworks used in RNA modification site prediction. **a)**. In the differential testing schemes, the input data is arranged by piling up the base called reads or extracted current signals along the reference sequence. The counts of mismatches or normalized current signals are summarized into contingency tables and subjected to categorical differential testing. **b)**. In the Bayesian models framework, either means or medians of the current signals covering each k-mer position are extracted as observed variables and are fitted by a Gaussian mixture model (GMM). The estimated weight parameter of the latent binomial variable can be regarded as the modification rate at the given k-mer. Differential methylation can be conducted over the quantified modification rate across samples to detect the presence of significantly differentially modified sites.

Differential statistical testing and Bayesian modeling are two major types of statistical methods used in RNA modification prediction from nanopore sequencing. The input of differential testing depends on the site-level information, which is often the coverage data of the base called reads or the extracted current signals mapped at the reference sequence. The sites level information is often summarized into contingency tables and subjected to statistical testing (Fig. 2a). Bayesian modeling act on the read-level information which is means or medians of the current signals of the base events in reads. Bayesian generative models such as Gaussian mixture model (GMM) will be fitted to the signal to detect signal shifts. In case of GMM, the posterior estimates for each mixture component can interpreted as the probability of modification at the given base (Fig. 2b).

#### 2.1.1 Differential testing

DiffErr [33] and DRUMMER [39] are two prediction methods adopting G-tests under the differential testing scheme. DiffErr predicts m^6^A modifications by finding differences in the base calling error rate between the target sample and the reference control sample. The input data is the stacked read coverage at the potential modification site labeled by base call errors. Specifically, the counts of five bases called results (A, C, G, U, or indel) are aggregated from each sample. An n × 5 contingency table is then generated for each base, where n denotes the number of samples and 5 represents the types of base calling results. A G-test is performed on this table to identify any base with a significant change in error rates. An additional G-test is performed on significant bases between other pairings of replicates in the same condition. Benjamini-Hochberg method with an 0.05 FDR threshold is used for multiple testing corrections. If the sum of the G statistics in the lateral G-test is larger than the G statistic in the former one, the base will be excluded from the candidate sites to improve stringency. Subsequently, the log2 fold changes of mismatch to match ratio at the candidate sites between different-conditioned samples are calculated. A base with a log2 fold change >1 is considered a modification site. DiffErr was tested on the nanopore direct RNA sequencing (DRS) data of Arabidopsis. The result showed that the m^6^A modification sites revealed by DiffErr are enriched in 3′ UTRs but not enriched around stop codons, which is consistent with the pattern of the paired miCLIP sample. About 66 % of the m^6^A modification sites discovered located within 5 nucleotides of miCLIP sites.

The testing method used by DRUMMER is also G-test on a 2 × 5 contingency table from the counts of basecall information for each base. A Bonferroni approach is used to adjust multiple-hypothesis testing. The two rows in the contingency table represent the methyltransferase knockout samples versus the wild-type samples. The fold change of mismatch to match ratio is then calculated at each included base. Candidate modification sites are selected if the site has a onefold or greater change and the adjusted G-test p-value is less than 0.01. This algorithm was tested on the nanopore DRS data of the wild-type (WT) and METTL3-knockout (M3KO) A549 cells infected with adenovirus serotype 5 (Ad5) with matching MeRIP-Seq data. Their results showed that approximately 83.1 % of the universally predicted sites across all datasets were located on AC motifs, with the remaining sites located within four nucleotides away from AC motifs. The observed distances were much smaller than random shuffled sites, suggesting a positive motif association. The sequence logo generated from the context of these candidate sites resembled the commonly known m^6^A DRACH motif. The predicted modification sites also overlapped extensively with peaks detected by MeRIP-Seq.

ELIGOS [36] uses Fisher’s exact tests to predict modified bases by comparing the base-call error profiles between the modified and control DRS data. The training set consisted of in vitro transcribed (IVT) sequence data with all target bases modified, a control IVT sample with no base modified, and a cDNA sample reversely transcribed from the native RNA sequences called the RNA background error model (rBEM). To apply a differential test, ELIGOS calculates the sum of mismatches, insertions, and deletions at each base of the reference sequence using the aligned reads. These features are referred to as the Error of Specific Bases (ESB). The differences in the proportion ESB (%ESB) between native RNA and the reference sequence data constitute three 2 × 2 contingency tables, one for each reference data. Fisher's exact tests is then performed and the q-values are computed by Benjamini-Hochberg correction. The methylation is called using user defined cut-offs for q-values and odds ratios. ELIGOS was tested on Curlcakes and IVT datasets having modification types from m^6^A, m^1^A, ^5^moU, Psi, m^7^G, Ino, hm^5^C, and f^5^C. The results showed that the best AUROC for Curlcakes data is 0.758 on all possible 5-mers and 0.973 on DRACH motifs. All the IVT data had AUROC >0.74 except for the one containing m5C modification. In addition, ELIGOS was further validated on DRS data of rRNAs from human cells, yeasts, and E. coli. The resulting AUROC were 0.895–0.938, 0.861–0.962, and 0.862–0.953, respectively.

#### 2.1.2 Bayesian modeling

Nanocompore [45], xPore [26], and Yanocomp [41] applied Bayesian generative models to predict the modification sites while supporting the methylation level quantification. Nanocompore predicts m^6^A modification sites by comparative analysis between experimental and control datasets. The median signal intensity and the log10 of dwell time of each read are extracted from raw current signals and used as input data. Pairwise comparisons are conducted between modified and control samples on all the possible 5-mers by the robust univariate test (Kolmogorov-Smirnov test) or bivariate Gaussian mixture model (GMM) with a logistic regression test. Nanocompore was first evaluated on in-silico generated data by simulating modified and unmodified current signals. This simulation was based on the current distribution from an IVT human DRS dataset. The unmodified current signals are mimicked by a probability density generator. The simulation of current signals for modified bases is generated by shifting the mean of density distribution. The evaluation results showed that the KS test has the highest sensitivity but with the expense of lower specificity, while the GMM models reach the lowest False Positive Rate and the highest F1 score. GMM models outperformed KS tests when the analysed transcripts had different sequencing depths and were therefore used in the following analysis. In a cell line independent validation test, Nanocompore was applied on the nanopore RNA sequencing data of shRNA-mediated METTL3 knock-down (K_D_) MOLM13 cell line and wild type (WT) MOLM13 cell line. The results showed that the m^6^A modification sites predicted were enriched near the stop codons of mRNA, resembling the pattern previously reported by the METTL3-dependent m^6^A sites. Compared with the miCLIP data of the MOLM3 cell line, 54 % of the modification sites predicted by Nanocompore overlapped with the miCLIP in WT cells and there exists a significant decrease in Nanocompore predicted modification sites at miCLIP crosslink sites. The methylation ratios at these sites are also significantly reduced from the WT to the K_D_ cell line. Although Nanocompore has only been tested and validated only on m^6^A modification, it is potentially applicable to any modification detection with proper control samples free of modification.

xPore predicts m^6^A modification sites through a multi-sample Bayesian Gaussian mixture model. The model takes input from the normalized mean of the current signals from all reads across different samples at each possible 5-mer. The two components of the mixed distributions are used to denote the unmodified and modified RNA species. The cluster closer to the theoretical distribution for unmodified RNA species is considered unmodified, while the other is modified. A Bayes classifier is used on the posterior probability of the soft clustering to predict modification events at the read level. The modification rate for a given site is estimated by the proportion of modified reads aligned at that position. xPore was tested on the nanopore DRS data of wild-type (WT) and METTL3 knockout (KO) HEK293T cell lines with matched m^6^ACE-Seq data as reference labels. Differential analysis was then applied to the estimated modification rates of replicates between WT and KO to determine whether the site is modified. Benjamini-Hochberg method with a 0.05 FDR threshold is utilized to adjust the multiple testing P-values. At the A-centered positions (NNANN), xPore achieved an AUROC of 0.86 and a precision of 0.6. The low precision could be a result of detecting modifications that were missed by antibody-based prediction. Over 90 % of the modification positions predicted with a p-value lower than 0.001 overlapped the modified DRACH motifs identified by m6ACE-Seq. The predicted sites formed a distribution peak near the stop codon, resembling the pattern identified by m6ACE-Seq. xPore also demonstrated a positive correlation between the quantified m^6^A levels in the HEK293T cell lines and the profiles acquired from MAZTER-Seq and m6ACE-Seq, with Spearman's rank correlation coefficient equal to 0.49 and 0.66, respectively. xPore has an advantage over other methods in that it can obtain the quantitative m^6^A levels from individual DRS experiments without depending on the associated KO samples.

Yanocomp constructs GMM based on the mean current values of each kmer at each read. The model contains two Gaussian components and one uniform component. The weights for the two Gaussian components are also utilized to estimate the modification rate at each position, followed by a G-test as the differential analysis between control and experiment samples. This algorithm was tested on the DRS data of Arabidopsis defective in the function of VIRILIZER (vir-1) and VIR complemented lines (VIRc). The average modification rates dropped by 81.8 % in the defective cell line compared to the WT condition. Most of the m^6^A modification sites identified were located at m^6^A consensus motifs and almost all the modification sites predicted were in 3′UTRs. Compared with miCLIP data, 84.1 % of the predicted modification sites were located within 5nt of the nearest miCLIP site. The authors also intend to improve Yanocomp to enable the prediction without the need for paired KO experiments.

### 2.2 Supervised learning

The supervised learning-based prediction methods often use heterogeneous input information, in which both the current intensity and the base called sequence are input features. This can often provide a performance advantage over statistical methods which typically use only one feature type. The adopted models or algorithms include K-means [38], K-nearest-neighbour [38], Random Forests [34], [42], Support Vector Machine [21], [31], [42], XGBoost [37] and neural networks [32], [40], [42], [43], [44].

Among these machine learning-based methods, EpiNano-SVM [21], [31], MINES [34], and nanoDoc [40] can only reach site-level predictions. EpiNano-SVM uses a Support Vector Machine (SVM) to classify the m^6^A-modified bases based on systematic base-calling errors in DRS datasets. The features are composed of per-site and per-kmer quality, mismatch, insertion, deletion, and current intensity. It can either uses absolute feature values or differences in feature values between two comparing samples. The SVM model was trained and tested on Curlcakes datasets with features of per-base quality, mismatch, and deletion at the middle position of the RRACH k-mers. The Curlcakes datasets, originally designed and generated by the authors of EpiNano, consist of the DRS data of the modified and control IVT sequences containing all possible 5-mers. Two biological replicates were generated for both modified and control datasets. The SVM model was trained on the 1st replicate of Curlcake datasets and tested on the 2nd replicate. The results showed that the AUROC were higher than 0.969. For cell line independent validation, the model was trained and tested on the nanopore DRS data of polyA(+)-selected RNA from wild-type (WT) and ime4 knockout (KO) yeast strains with distinct subsets of features. The model reached the AUROC higher than 0.613 with absolute feature values and 0.636 with differences in feature values. In the recent update EpiNano 1.2, the authors implemented another mode named EpiNano-Error, which predicts modification sites by testing for differences in feature values between two comparing samples. Z-score deviances and data point residuals after fitting the linear regression models are used to determine the modification sites. The Bonferroni method is used to correct for multiple testing p-values. The threshold parameters can be user-defined.

MINES classifies the m^6^A sites on DRACH motifs through a random forest model using the features of modification stoichiometries within a 20-bp region. The centre position (Position 0) of the region is an adenine nucleotide in the middle of the DRACH motifs. The modification stoichiometries are calculated by Tombo by averaging the modification probabilities of each read. The random forest models were trained and tested on the HEK293T cell line using labels from matched m6ACLIP-seq datasets. The DRACH motifs containing overlaps with m^6^A modification sites in label datasets were considered as positive sites and otherwise negative sites. The obtained results reached the accuracies ranging from 67 % to 83 %, the precisions ranging from 40 % to 92 % and the AUROC ranging from 54 % to 76 % for all 18 models over instances of DRACH motif. Among them, only 4 models (the ones for the AGACT, GGACA, GGACC, and GGACT motifs) had AUROC beyond 0.67. In the independent validation on the HMEC cell line, the m^6^A sites predicted by MINES were enriched near the start of 3′UTR across all transcript isoforms, which resembled the results from typical m6A-seq. In the future, MINES may upgrade to realize read-level prediction.

nanoDoc distinguishes RNA modifications at the site level through a combination of deep learning and machine learning. It adopts a Deep One-Class (DOC) classification model with transfer learning and takes normalized current signals and the dwell time of each possible 5-mer as the input feature. Two parallel DOC classification models are constructed to calculate the Euclidean distance between the output vectors of target 5-mers in the secondary network and output vector of other 48 similar 5-mers in the reference network. The obtained Euclidean distances constitute a distribution for each 5-mers. The reference network was trained on IVT data free of modification. Besides, the IVT data was also used as the control dataset to calculate the distance distributions. During the application of nanoDoc, two pairs of parallel DOC classification models were processed simultaneously. The distribution of distances between two IVT read datasets (control distribution) and the distribution between an IVT read dataset and a native sequence read dataset (native distribution) were generated, respectively. Next, these two distributions were scored through cumulative distribution functions. With the modification present, the score of the control distribution was expected to be greater than that for the native distribution. To distinguish between different classifications, the native sequence 5-mer data were processed through a reference classification network followed by K-means clustering. nanoDoc was tested on the nanopore DRS data of native rRNA of E. coli and yeast with matched reference IVT sequencing data. It tried to detect all 23 kinds of RNA modification and achieved an AUROC of 0.96. Within 7 5-mers, 10 out of 15 types of modifications were successfully identified with>50 % specificity. nanoDoc was also applied on Curlcakes data to predict m^6^A sites and it achieved an AUROC of 0.68 on all possible 5-mers. Most modification sites within DRACH motifs were successfully detected. Currently, nanoDoc cannot perform read-level predictions for modification rate quantification. In the future, nanoDoc will accumulate more data regarding different modification types in the 5-mers and will be independent of the IVT reference data.

NanoRMS [38], nanom6A [37], and Penguin [42] implement read-level prediction using classic machine learning algorithms such as Random Forest (RF), Support Vector Machine (SVM), and K-Nearest-Neighbourhood (KNN). NanoRMS predicts the modification sites and modification levels of pseudouridine (psi) and Nm modification via either unsupervised (K-means) or supervised (KNN) machine learning algorithms. The authors use combined input features of signal intensity calculated from Tombo and base probability (trace) reported from Guppy. NanoRMS was trained and tested on the yeast strains of wild-type (WT) and two different knockouts for psi and Nm modification, respectively. The predicted per-site modification stoichiometries were verified by mass spectrometry results. Significant reductions in predicted stoichiometries were observed at positions specific to one of the psi and Nm-modified snRNA-depleting cell lines.. NanoRMS was also validated on data of the poly(A)-selected RNA from wild-type (WT), Pus1 knockout, and Pus4 knockout yeast strains. The predictions successfully recapitulated 11 % and 75 % of previously reported Pus1- and Pus4-dependent psi modification sites, respectively. NanoRMS also discovered some novel modification sites across replicates. The results revealed similar mismatch patterns to previously reported psi modification sites, and the predicted sites were predominantly responsive to the Pus1 and Pus4 knockout.

Nanom6A uses the XGBoost model to predict m^6^A modification sites at RRACH motifs. The input features of the XGBoost model include the median, standard deviation, mean, and dwell time of the current signals over each RRACH motif extracted by the Tombo re-squiggle function. Nanom6A was trained and tested on the Curlcakes dataset. The authors obtained an AUROC of 0.97 under 10-fold cross-validation, and approximately 91–96 % of modification sites are uncovered. For cell line independent validation, the prediction over the wild type (WT) and METTLE3 knockdown (K_D_) HEK293T cell line were compared with the matched SCARLET and MeRIP-Seq data. In addition, the Arabidopsis mutant defective in the function of VIRILIZER (vir-1) and VIR complemented lines (VIRc) and stem-differentiating xylem (SDX) of Populus trichocarpa with matched MeRIP-Seq and m6A-REF-seq data were also compared. In the HEK293 cell line, the numbers of predicted m^6^A modification sites and the predicted modification rates showed a decline in K_D_ samples at ACTB sites, resembling the results reported from SCARLET and MeRIP-Seq. In the Arabidopsis cell line, 40 % of the m^6^A modification sites predicted were shared with those detected by EpiNano or MINES, and 66 % of the RRACH motifs with predicted modification sites were also predicted by DiffErr. The number and modification ratios of m^6^A sites detected by Nanom6A dropped in the VIRc relative to the vir-1. In the Populus trichocarpa, Nanom6A predicted m^6^A with a similar enrichment pattern compared with the MeRIP-Seq and m6A-REF-seq. Approximately 81 % and 80 % of the modification sites predicted overlapped with MeRIP-Seq and m6A-REF-seq, respectively.

Penguin predicts pseudouridine modification at all possible 5-mers by assembling three models of Support Vector Machine (SVM), Random Forest (RF), and Neural Network (NN). It uses Nanopolish to extract current signal features as the model input, including the mean, standard deviation, dwell time of the current signals, and the k-mers of the events aligned to the reference genome. Penguin was trained and tested on the nanopore DRS data from the HEK293 cell line, with the matched benchmark dataset as labels. The obtained results showed that the SVM (Accuracy: 0.9338, Recall: 0.95, AUROC: 0.933, Precision: 0.92) had a higher accuracy, recall and AUROC and a lower precision compared to RF (Accuracy: 0.8459, Recall: 0.72, AUROC: 0.852, Precision: 0.98). The SVM also achieved comparative results with NN (Accuracy: 0.9335, Recall: 0.95, AUROC: 0.932, Precision: 0.92). In the cell line independent testing on the nanopore DRS data of HeLa cell line, NN (Accuracy: 0.9535, Recall: 1.00, AUROC: 0.953, Precision: 0.92) outperformed SVM (Accuracy: 0.9261, Recall: 0.94, AUROC: 0.926, Precision: 0.91) in all aspects. RF was excluded from the results due to its low accuracy (<0.5). Penguin uncovered pseudouridine modification sites from 7148 genes at 6482 unique genomic locations shared across the HEK293T and HeLa cell lines. Among the top 1 % frequent pseudouridine modification genomic locations in the HEK293T and HeLa cell lines, 15.8 % were commonly detected across both cell lines. In the future, Penguin may integrate advanced deep learning methods for modification prediction.

There are three methods using deep learning models for modification sites and quantification prediction, which are DENA [44], m6Anet [43], and nano-ID [32]. DENA predicts m^6^A modification on RRACH motifs through Bidirectional Long Short-Term Memory (Bi-LSTM) neural network. A total of 12 Bi-LSTM neural networks are constructed for each RRACH motif. It extracts the mean, median, standard deviation, dwell time, and base quality of the current signals at RRACH motifs as input features through the Tombo re-squiggle algorithm. DENA is the first neural-network-based RNA prediction tool trained on in vivo transcribed mRNA data. The network was trained and tested on the nanopore DRS data of Arabidopsis defective in the function of VIRILIZER (vir-1) and VIRILIZER complemented cell lines (VIRc). The AUROC and the accuracies of all the 12 models reached between 0.90 and 0.97 and between 0.83 and 0.93, respectively. For validation, DENA was applied on the wild-type (Col-0) Arabidopsis and full training dataset (vir-1 and VIRc). A large fraction of overlap was observed between the predicted modification sites in Col-0, VIRc, and vir-1 cell lines. A high correlation between modification rates was discovered between VIRc and Col-0. The m^6^A sites predicted were also enriched near the stop codon and 3′UTR, similar to the expected distribution of m^6^A modifications.

m6Anet is a prediction software based on Multiple Instance Learning. The model consists of two joined modules of a read-level encoder and a pooling layer. The encoder converts the input features into a high-dimensional representation, followed by the read-level prediction of modification rates with two hidden layers. The noisy-OR pooling layer integrates the read level prediction to estimate the probability of modification at the site level. The normalized mean, standard deviation, and dwell time of the nanopore raw signals of each read at each position (i) and the single position before (i − 1) and after (i + 1) are used as input features in this model. The sequence information of all possible 5-mers in the training data is encoded as a two-dimensional vector. The embedding is also used as an input feature for the model. The labels for the model are the binary modification status at each site marked with 1 (modified) or 0 (otherwise). The modification status can be observed from other sequencing methods such as m6ACE-Seq or other gold-standard data. The training data for m6Anet was the nanopore DRS data of the HCT116 cell line and the training labels were the matched m6ACE-Seq data. Sites mapped to non-DRACH motifs were all removed from training data. The trained m6Anet model was evaluated on the HEK293T cell line using the ground truth of the matched m6ACE-Seq and miCLIP data. The results showed an AUROC of 0.83 and a PRAUC of 0.35 on DRACH motifs, outperforming EpiNano and Tombo. m6Anet also achieved the AUROC of 0.83 and 0.83 and PRAUCs of 0.43 and 0.37 on 4 motifs (AGACT, GGACA, GGACC, and GGACT) and all RRACH motifs, respectively, outperforming MINES and nanom6A. m6Anet can generalize well to new DRS data without the requirement of new training data.

nano-ID predicts the sites of nucleoside analogues 5-Ethynyluridine (5EU) at the read level via a neural network model. 5EU is a chemical modification used to identify newly synthesized RNA, which is often generated through metabolic RNA labelling. The neural network takes the normalized raw current signals, base-calling event probability, and alignment mismatch properties as the input features. The network architecture is constituted of a batch normalization layer and two dropout layers between the three dense layers. nano-ID was trained on the nanopore DRS data of the myelogenous leukemia K562 cell line. The cells were exposed to 5EU labelling for 24 h (5EU 24 h), exposed to 5EU labelling for 60 min (5EU 60 min), or not exposed to 5EU labelling (Control). The trained network achieved an accuracy of 0.87 and an FDR of 0.025 on the 5EU 60 min cell line. The model achieved the AUROC of 0.94 overall and 0.96 for reads with lengths >500nt and 1000nt. Similar results were obtained from a random forest classification with the same training and testing data. Improvements of both the nanopore sequencing platform and the base calling algorithm are expected to further improve the accuracy of Nano-ID.

## 3 Discussion

Epitranscriptomics is a field that has been profoundly advanced in recent decades. The research momentum is mainly driven by the breakthrough of sequencing technologies that enabled the omic-level analysis of RNA modifications. With the recent development of third-generation sequencing technology, nanopore direct RNA sequencing has the potential to realize the base resolution detection of RNA modification over long native RNA sequence reads independent of sequencing biases introduced by PCR amplification and reserve transcription.

Here we have comprehensively reviewed 15 published RNA modification prediction tools based on direct RNA sequencing data. Among them, 6 use statistical models and 9 are based on supervised machine learning (Table 1). The principles behind these methods all depend on recognizing the perturbation of the signal intensity in modified bases that are uniquely associated with each k-mer context. One major difference in application is that the statistical testing-based methods often rely on the paired reference control dataset that has no modification, which is typically obtained from either IVT or the K_D_/KO experiment. In contrast, machine learning and deep learning models can generalize the signal pattern learned from the training data set without requiring the paired no-modification control sample.

**Table 1:** Comparison over 15 published tools.

	Modification	Species	AUROC	Resolution	Motif
**DENA**	m^6^A	Arabidopsis	0.90–0.97	Read	RRACH
**nanom6A**		Arabidopsis; Human; IVT; Populus trichocarpa	0.97	Read	
**EpiNano**		IVT; Yeast	–	Site	
**m6Anet**		Hunan	0.83	Read	DRACH
**MINES**		Human	0.54–0.76	Site	
**Nanocompore**		Human; IVT	0.9889- 0.9947	Site	All possible 5-mers
**xPore**		Human	0.86	Read	
**Yanocomp**		Arabidopsis	–	Read	
**DiffErr**		Arabidopsis	–	Site	
**DRUMMER**		Human	–	Site	
**nanoDoc**	Multiple modifications	Yeast; E. coli; IVT	Yeast and E. coli: 0.96 IVT: 0.68	Site	
**ELIGOS**		Human; IVT; Yeast; E. coli	IVT: > 0.74 Human: 0.895–0.938 E. coli: 0.861- 0.962 Yeast: 0.862- 0.953	Site	
**NanoRMS**		Yeast	–	Read	
**nano-ID**	5EU	Human	0.94	Read	
**Penguin**	Psi	Human	0.852–0.953	Read	

Regarding the supported modification types of the prediction methods, most of the methods (12/15) are trained and applied to only one type of modification (Fig. 2). However, almost all of their computational frameworks have the potential to apply to other modifications as long as providing a high-quality training set. However, one of the major limitations is that the current DRS data may not support the accurate distinction between certain kinds of modifications due to the overlap between their signal shifts, such as between ψ and m^1^ψ [46]. In addition, 7 out of 15 methods predict modification only at the site level without reporting the quantitative modification profile. The difference in their functionalities can be attributed to the limitations of computational frameworks and programming implementation.

Sequencing depth is also a strong factor influencing the detection power of modification sites, especially for the prediction tools based on statistical models. If the sequencing depth is too low, the differential testing can be significantly affected by the reduction of statistical power, especially considering the relatively low sequencing accuracy of Nanopore sequencing. Common ways of reducing the high statistical noise of low sequencing depths are pooling reads across replicates [44] or excluding sites below a coverage threshold [21], [33], [34], [35], [38], [39]. The default thresholds of minimum read coverage often vary between different tools (see Table 2).

**Table 2:** Overview of the features and the models used in the methods reviewed by this article. Feature level describes the type of input features used by the software. All methods modeled on the raw electrical current are classified into the signal intensity, and otherwise, it is labeled as the base call error.

	Feature level	Model/Method	URL
***DENA***	Signal intensity	Deep Learning	https://github.com/weir12/DENA
***m6Anet***			https://github.com/GoekeLab/m6anet
***nanoDoc***			https://github.com/uedaLabR/nanoDoc
***MINES***		Machine Learning	https://github.com/YeoLab/MINES.git
***nano-ID***			https://github.com/birdumbrella/nano-ID
***nanom6A***			https://github.com/gaoyubang/nanom6A
***NanoRMS***			https://github.com/novoalab/nanoRMS
***Penguin***			https://github.com/Janga-Lab/Penguin
***EpiNano-SVM***			https://github.com/novoalab/EpiNano
***Nanocompore***		Bayesian Modelling	https://github.com/tleonardi/nanocompore
***xPore***			https://github.com/GoekeLab/xpore
***Yanocomp***			http://www.github.com/bartongroup/yanocomp
***DiffErr***	Base call error	Differential Tests	https://github.com/bartongroup/differr_nanopore_DRS
***DRUMMER***			https://github.com/DepledgeLab/DRUMMER
***ELIGOS***			https://gitlab.com/piroonj/eligos2
***EpiNano-Error***			https://github.com/novoalab/EpiNano

Furthermore, all the currently reported methods are not free of false positive methylation calls due to various reasons. Most of such false positivity may be attributed to the overfitting caused by the confounded training samples. Besides, the low differentiability of the electrical signal under certain k-mer contexts may inevitably introduce the sequence-dependent prediction error. Some signal shifts resulting from modifications are observed near the true modification sites, which are also likely to introduce false positive calling. One possible approach to lowering false positivity is to narrow the candidate sites only on consensus motifs such as DRACH for m^6^A. Mistakes generated from low-quality reads and poor alignment results are also potential causes of false positivity. In the framework of Bayesian models, one way to reduce false positives is to only consider signal shifts in one direction. Eventually, for all the computational frameworks, a trade-off is always expected between accuracy and precision upon different cutoffs of predicted values. Therefore, many variations between the prediction outcomes of different methods can be attributed to the difference in the stringencies of the default thresholds.

**Fig. 3:**
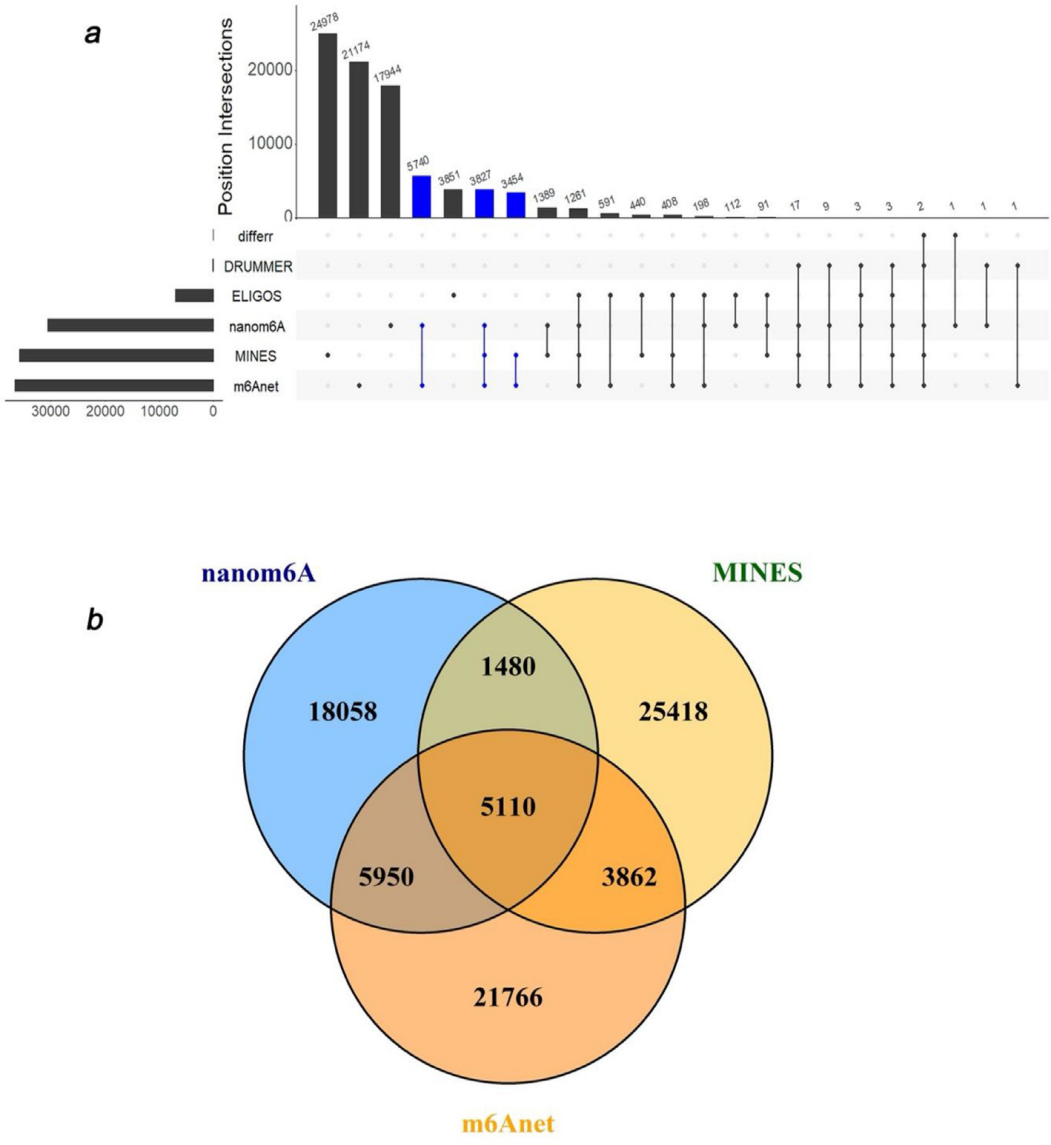
The comparison of the default m^6^A sites called from different tools. **a)**. The upset plot shows the number of sites predicted by DiffErr, DRUMMER, ELIGOS, nanom6A, MINES, and m6Anet and their overlaps with each other. **b)**. The Venn diagram of predicted sites from the top three consistent methods: nanom6A, MINES, and m6Anet.

Multiple previous studies have tried to make comparisons between different tools [35], [37], [43], [44], [47]. Here, we also conducted a primary comparison between DiffErr, DRUMMER, ELIGOS, nanom6A, MINES, and m6Anet on a single fast 5 data (GSM3897645; HEK293T WT) [34]. All tools were run using default settings. Interestingly, sites predicted by DiffErr and DRUMMER have extremely poor overlaps with the other four methods (Fig. 3a). Both two methods have adopted statistical testing as their prediction schemes. In contrast, nanom6A, MINES, and m6Anet have made relatively consistent predictions (Fig. 3a), with 5110 sites commonly reported by all three methods (Fig. 3b). To be noticed, the three methods all use the machine learning framework. This primary comparison revealed that the prediction made by different tools are likely to exhibit considerable variations, and the computational schemes adopted may contribute significantly to the differences. Consequently, a systematic evaluation of the performances of different tools is necessary for the field. The codes for this comparison are available at https://github.com/XichenZhao0223/Nanopore-tools-comparison. There is also a call for defining robust evaluation metrics and strong benchmark datasets, as most currently published tools have used different pre-processing and evaluation schemes (Table 3) (Table 4).

**Table 3:** Overview of the capabilities of reviewed methods. The modification column only records the modification used in the training data within the initial publication. The species column summarizes the species of the data used for training, testing, and validation. The coverage filter records the minimum sequencing depths required for some tools on specific sequencing datasets, which can also be a user-defined parameter. The AUROC column records the performance of each method under different benchmark datasets. The level is indicating whether the software can predict the modification in individual reads or not. The motif column summarizes the scope of the 5-mer motifs applied as input features during modelling.

	Modification	Species (Coverage filter)	AUROC	Level	Motif
***DENA***	m^6^A	*Arabidopsis*	0.90–0.97	Read	RRACH
***nanom6A***		*Arabidopsis*; Human; IVT; *Populus trichocarpa*	0.97	Read	
***EpiNano-SVM***		IVT; Yeast (>5 reads)	IVT: > 0.969 Yeast: 0.613–0.693	Site	
***m6Anet***		Human	0.83	Read	DRACH
***MINES***		Human (>5 reads)	0.54–0.76	Site	
***Nanocompore***		Human (>30x); IVT	0.9889–0.9947	Site	All possible 5-mers
***xPore***		Human	0.86	Read	
***Yanocomp***		*Arabidopsis*	Not mentioned	Read	
***DiffErr***		*Arabidopsis* (>10 reads)	Not mentioned	Site	
***DRUMMER***		Human (>100x)	Not mentioned	Site	
***nanoDoc***	Multiple modifications	Yeast; *E. coli*; IVT	Yeast and E. coli: 0.96 IVT: 0.68	Site	
***ELIGOS***		Human; IVT; Yeast; *E. coli*	IVT: > 0.74 Human: 0.895–0.938 E. coli: 0.861–0.962 Yeast: 0.862–0.953	Site	
***NanoRMS***		Yeast (>30 reads)	Not mentioned	Read	
***nano-ID***	5EU	Human	0.94	Read	
***Penguin***	Psi	Human	0.852–0.953	Read

**Table 4:** Preprocessing schemes used in reviewed methods. DiffErr, DRUMMER, ELIGOS, and EpiNano only use features of the base call error; thus, no feature extraction software is applied by these methods.

The breakthrough in the accuracy of nanopore-based modification prediction methods may eventually rely on two factors: 1.) the preparation of high-quality, unconfounded IVT training data. 2.) the upgrade of the 3rd generation sequencing platform that provides more differentiability between signals of modified and normal bases. Meanwhile, the new computational models can be improved both in accuracy and computational efficiency (fast and small memory demands). If the above criteria have been met, the refined computational tools can eventually enable the detection of all modification types simultaneously within a single RNA molecule. Such detection power can shed light on important epitranscriptomic phenomena, such as revealing the interaction between different modification types on a single molecular transcript. From a long-term perspective, RNA modification detection based on direct RNA sequencing is a promising novel technique to improve our understanding of epitranscriptomic.

## Declaration of Competing Interest

The authors declare that they have no known competing financial interests or personal relationships that could have appeared to influence the work reported in this paper.
